# Comparative expression analysis of the *Atoh7* gene regulatory network in the mouse and chicken auditory hindbrain

**DOI:** 10.1007/s00441-023-03763-9

**Published:** 2023-03-24

**Authors:** Ali Jason Saleh, Yannis Ahmed, Lars-Oliver Peters, Hans Gerd Nothwang

**Affiliations:** 1 Division of Neurogenetics and Cluster of Excellence “Hearing4all”, School of Medicine and Health Sciences, Carl von Ossietzky University Oldenburg, 26111 Oldenburg, Germany; 2 Institute of Neurophysiopathology (INP), Aix-Marseille University, CNRS, Marseille, France; 3 Research Center for Neurosensory Science, Carl von Ossietzky University Oldenburg, 26111 Oldenburg, Germany; 4 Department of Neuroscience, Cluster of Excellence “Hearing4all”, Carl von Ossietzky University Oldenburg, 26111 Oldenburg, Germany

**Keywords:** Auditory system, Vertebrate, Gene regulatory network, Gene expression, Evolutionary development

## Abstract

The mammalian and avian auditory brainstem likely arose by independent evolution. To compare the underlying molecular mechanisms, we focused on *Atoh7*, as its expression pattern in the mammalian hindbrain is restricted to bushy cells in the ventral cochlear nucleus. We thereby took advantage of an *Atoh7* centered gene regulatory network (GRN) in the retina including upstream regulators, *Hes1* and *Pax6,* and downstream targets, *Ebf3* and *Eya2*. In situ hybridization demonstrated for the latter four genes broad expression in all three murine cochlear nuclei at postnatal days (P) 4 and P30, contrasting the restricted expression of *Atoh7*. In chicken, all five transcription factors were expressed in all auditory hindbrain nuclei at embryonic day (E) 13 and P14. Notably, all five genes showed graded expression in the embryonic nucleus magnocellularis (NM). *Atoh7* was highly expressed in caudally located neurons, whereas the other four transcription factors were highly expressed in rostrally located neurons. Thus, *Atoh7* shows a strikingly different expression between the mammalian and avian auditory hindbrain. This together with the consistent absence of graded expression of GRN components in developing mammalian nuclei provide the first molecular support to the current view of convergent evolution as a major mechanism in the amniote auditory hindbrain. The graded expression of five transcription factors specifically in the developing NM confirms this nucleus as a central organizer of tonotopic features in birds. Finally, the expression of all five retinal GRN components in the auditory system suggests co-options of genes for development of sensory systems of distinct modalities.

**Supplementary Information:**

The online version contains supplementary material available at 10.1007/s00441-023-03763-9.

## Introduction

Both the mammalian and avian auditory brainstem contain a variety of interconnected nuclei with morphologically corresponding neurons. This resemblance together with other features such as similarities in neural circuit organization and function resulted in the early view that some nuclei and circuits are homologous (see (Grothe et al. 2004) for a critical review). In agreement, both the mammalian and avian second auditory nuclei were often referred to as the cochlear nuclei (Sullivan and Konishi 1984; Raman and Trussell 1992; Jackson and Parks 1982). However, current perspective refutes true homology between tetrapod central auditory nuclei for two main reasons: i) Independent development of ears with a tympanic membrane among several terrestrial tetrapod groups, including mammalian and avian ancestors (Clack 1997, 2002; Kitazawa et al. 2015). ii) Major differences in topological positions, electrophysiological properties, projection and wiring patterns, and computational mechanisms in the central auditory system (Grothe et al. 2004; Nothwang 2016). Current view therefore holds that the hindbrain nuclei of mammals and birds represent homoplasious structures, i.e. similar structures arisen by independent evolution. Their morphological and physiological similarities thus reflect similar constraints in detecting airborne sound after the transition of tetrapods from water to land (Clack 2012). Homoplasies can emerge via convergent and parallel evolution, depending on the molecular mechanisms. Their arrival by different developmental mechanisms is called convergent evolution, whereas they appear through parallel evolution when they share similar, perhaps identical, developmental pathways (Hall 2003).

Recent comparative analyses revealed similar broad expression patterns of transcription factors and evolutionary more dynamic microRNAs in the amniote auditory brainstem (Pawlik et al. 2016; Krohs et al. 2021; Saleh and Nothwang 2021), pointing to parallel evolution. The reported differences in the amniote auditory brainstem as well as conceptual evolutionary arguments argue, however, for conspicuous differences in GRN components as well and thus for convergent evolution (Nothwang 2016; Grothe and Pecka 2014). Highly selectively expressed genes might represent attractive candidates for such clade-specific features. We hypothesized that the basic helix-loop-helix (bHLH) transcription factor *Atoh7* may represent such a factor. In the mammalian hindbrain, *Atoh7* is specifically expressed in bushy cells of the ventral cochlear nucleus (VCN) (Saul et al. 2008), whereas its expression in the avian hindbrain is unexplored. Of note, *Atoh7* deficient mice display smaller bushy cells and altered auditory brainstem responses suggesting a critical role of this transcription factor for proper development of the auditory brainstem (Saul et al. 2008).

Extensive studies in retinal ganglion cells provided insight into an essential GRN required for their formation with *Atoh7* in the core, *Hes1* and *Pax6* acting as upstream inhibitor and activator, respectively, and *Ebf3* and *Eya2* as downstream targets (Mu et al. 2008; Gao et al. 2014; Wu et al. 2015). Thus, an entire GRN module can be analyzed for conservation in the amniote auditory brainstem. Furthermore, comparison between the auditory and visual system will provide insight into co-option of circuit assembly mechanisms across sensory systems (Sitko and Goodrich 2021). We therefore performed a comparative developmental expression analysis of the *Atoh7* centered GRN in the murine and chicken hindbrain. This identified for the first time a striking difference in the expression of a GRN component between the mammalian and avian auditory hindbrain, thereby supporting the view of their convergent evolution.

## Materials and methods

### Animals

Lohmann Brown chicks (*Gallus gallus*) at E13 (Hamburger-Hamilton stage 39), and P14 of both sexes were used (Hamburger and Hamilton 1951). NMRI mice (*mus musculus*) at P4 and P30 of both sexes were used. In both species, the selected stages included thus one prehearing stage and one after hearing-onset. All protocols were approved by the local animal care and use committee (LAVES, Oldenburg). All experiments were in accordance with the regulations of the German federal law on the care and use of laboratory animals and followed the guidelines of EU Directive 2010/63/EU for animal experiments. Fertilized eggs were kindly provided by a local farm (Geflügel-Siemers GmbH & Co. KG, Lohne, Germany) and were incubated at 38 °C in a humidified incubator.

### RNA probe preparation

RNA probe generation was conducted similarly to previous studies (Pawlik et al. 2016; Krohs et al. 2021; Saleh and Nothwang 2021). Probes for *Atoh7, Hes1, Pax6, Ebf3, and Eya2* were prepared from PCR amplification of fragments in the chicken and mouse genomic DNA pertaining to the relevant transcription factor gene with the corresponding primer pairs (Table 1). PCR amplicons ranging from 240 to 570 nt were cloned into the pGEMT-Easy vector (Promega, Walldorf, Germany). In vitro transcription of sequence verified clones with the SP6 and T7 polymerase in presence of digoxigenin-11-UTP (Roche Diagnostics, Manheim, Germany) resulted in Digoxigenin-labeled sense and antisense RNA probes, respectively. RNA probes were further purified with the RNeasy Mini Kit (QIAGEN, Hilden, Germany). The sense probes served as negative control yielding no staining (data not shown), whereas the antisense probes did. Specificity of the antisense probes was further indicated by the different expression patterns observed for each of the selected genes.

**Table 1:** Primers used in RNA probe generation

**Gene**	**NCBI accession**	**Forward primer** (5´ →3´)	**Reverse primer** (5´ → 3´)	**Amplicon size**
Chicken				
*Atoh7*	NC_052537.1	CCAGTCATTTGGATTCAGGACTAG	TGTCTGTTGCCACTTTTTGTCC	378 bp
*Hes1*	NC_052540.1	TGTACGGTGGTTTCCAGCTG	AACACGAAACACTGTCGGAG	312 bp
*Pax6*	NC_052536.1	CTGGACTGTCAGTTCCAGTTC	CCGATATAATGCCTTCAGTG	245 bp
*Ebf3*	NC_052537.1	ACGCATCAAACTGGAAGAAG	GTGTTGCGATGGGTAAGACT	549 bp
*Eya2*	NC_052551.1	CAGTGGAAGCCTCTAAGGAC	TTTGCAGAAGGTCCGACGTG	276 bp
Mouse				
*Atoh7*	NC_000076.7	AAGCTGTCCAAGTACGAGACACTGC	GTTTCTCCACCTCCTGAATGACGCT	567 bp
*Hes1*	NC_000082.7	CACCCTGCAAGTTGGGCAG	GTCTCTCCTAAAATCCAAGTTC	331 bp
*Pax6*	NC_000068.8	TGTAGGTCTGCATCCCACAAT	AAGGCCTTTAACTCCCACCG	464 bp
*Ebf3*	NC_000073.7	GGTCAGATCCCACAGCACTG	GAGGACACTTGCTAATGTGC	460 bp
*Eya2*	NC_000068.8	TTTCTGGAGGATATCCTGTC	ACGCACACTGTCCACATGAC	481 bp

### Brain slice preparation

Embryos and chicks, as well as mice were injected with a lethal dose of sodium pentobarbital (Narcoren©, 16 g/100 ml, Boehringer Ingelheim Vetmedica GmbH, Ingelheim, Germany; 2.5 ml/kg bodyweight). Chicken embryos were decapitated and heads were fixed by immersion in 4% PFA [4% paraformaldehyde in phosphate buffered saline (PBS, 136.9 mM NaCl, 2.7 mM KCl, 10.1 mM Na_2_HPO_4_, 1.8 mM KH_2_PO_4_), pH 7.4] at room temperature for ~ 4 h. Chicks and mice were perfused transcardially with phosphate buffered saline [PBS, 136.9 mM NaCl, 2.7 mM KCl, 10.1 mM Na_2_HPO_4_, 1.8 mM KH_2_PO_4_, pH 7.4] followed by 4% PFA [4% paraformaldehyde in PBS, pH 7.4]. Brains were postfixed in 4% PFA for 4–6 h and incubated for at least 24 h in 30% sucrose in PBS. Brains were embedded in Tissue Freezing Medium (General Data Healthcare, Cincinnati, Ohio, USA) and stored at -20 or -80 °C until slicing. Coronal sections of 25 µm thickness were cut on a Cryostat (Leica Biosystems, Nußloch, Germany) and stored at -80 °C.

### On-slide RNA in situ hybridization

On-slide sections were washed with PBS (+ 1% Tween) before being treated with proteinase K (10 µg/ml, Carl Roth, Karlsruhe, Germany) for 4–5 min (embryonic chicken and pup sections) or for 8 min (young chick and mice sections) and acetylated for 10 min [12.5 µl acetic anhydride in 5 ml 0.1 M triethanolamine in 0.9% NaCl]. Slices were then incubated 2—4 h at 50 °C in hybridization buffer [50% formamide, 5 × SSC, 2% Roche blocking reagent (Roche Applied Science, Penzberg, Germany), 0.02% SDS, 0.1% N-lauryl-sarcosine], followed by an overnight incubation at 50 °C in hybridization buffer containing 1 µg/ml RNA probe. After 3 consecutive washes for 30 min each at 45 °C with 2 × SSC, 0.5 × SSC, and PBS (+ 1% Tween), slices were incubated for 1 h with blocking solution [1% blocking reagent (Roche Applied Science)] in maleic acid buffer [0.1 M maleic acid, 0.15 M NaCl, pH 7.5] at room temperature (RT) followed up by a 1.5 h incubation with an alkaline phosphatase (AP) conjugated antibody against digoxigenin (Anti-DIG AP, Roche Applied Science, Roche Cat# 11093274910) in a 1:1000 dilution in blocking solution. Signal detection was performed in presence of NBT/BCIP staining solution (Roche Applied Science) 1:50 in AP-Buffer [100 mM Tris, 150 mM NaCl, 5 mM MgCl_2_, pH 9.5] at RT. In situ hybridization was repeated at least three times for each probe on at least three different animals. Images shown are representative results.

### Microscopy and image processing

Results were documented with an Olympus (DP74) camera installed on an upright Olympus microscope (BX63) (Olympus Deutschland, Hamburg, Germany) using the cellSens imaging software (Olympus Soft Imaging Solutions, Münster, Germany). Images were further processed for enhanced visualization and scale bar addition using ImageJ (FIJI) software (https://fiji.sc/) (Schindelin et al. 2012). Macros were written to automate all image processing steps.

### Quantitative expression analysis

Equal sized regions of interest (ROI) from different rostro-caudal regions of the embryonic NM were selected for quantification (Fig. 7b). For each coronal slice a region from the background tissue with the same size of selected ROIs was chosen for intensity normalization. Grayscale images were inverted for intensity measurements, so that regions with highest transcription factor expression were the brightest (Fig. 7d). The mean gray value of each ROI was calculated by ImageJ (FIJI) and two to four replicates were measured for each ROI. Graphpad Prism 9.4.1 (GraphPad, San Diego,CA) was used for statistical analysis. Unpaired t-test was used to analyze the statistical difference in the means of intensity between compared groups in this study. P-values < 0.05 were considered significant.

A decision tree was designed to quantitively categorize the different observed patterns of transcription factor expression in the NM (Fig. 7a). Homogeneous expression is considered when the expression intensity in the rostral regions was not significantly different from that in the caudal regions. If the expression intensity between the rostral regions (average of rostro-medial and rostro-lateral ROIs) and caudal regions (average of caudo-medial and caudo-lateral ROIs) showed a significant difference, the expression pattern was considered differential. Some differential expression patterns were further categorized into gradient or selective. The gradient pattern is characterized by having a significant decrease in expression across the middle NM sections from the medial (MM) to the central (MC) and further on from the MC to the lateral region (ML). The selective pattern shows only a significant difference between the MM and MC but no significant change from the MC to the ML.

## Results

### Spatiotemporal expression pattern of *Atoh7* related transcription factors in the developing auditory hindbrain of mice and chicken

**Fig. 1:**
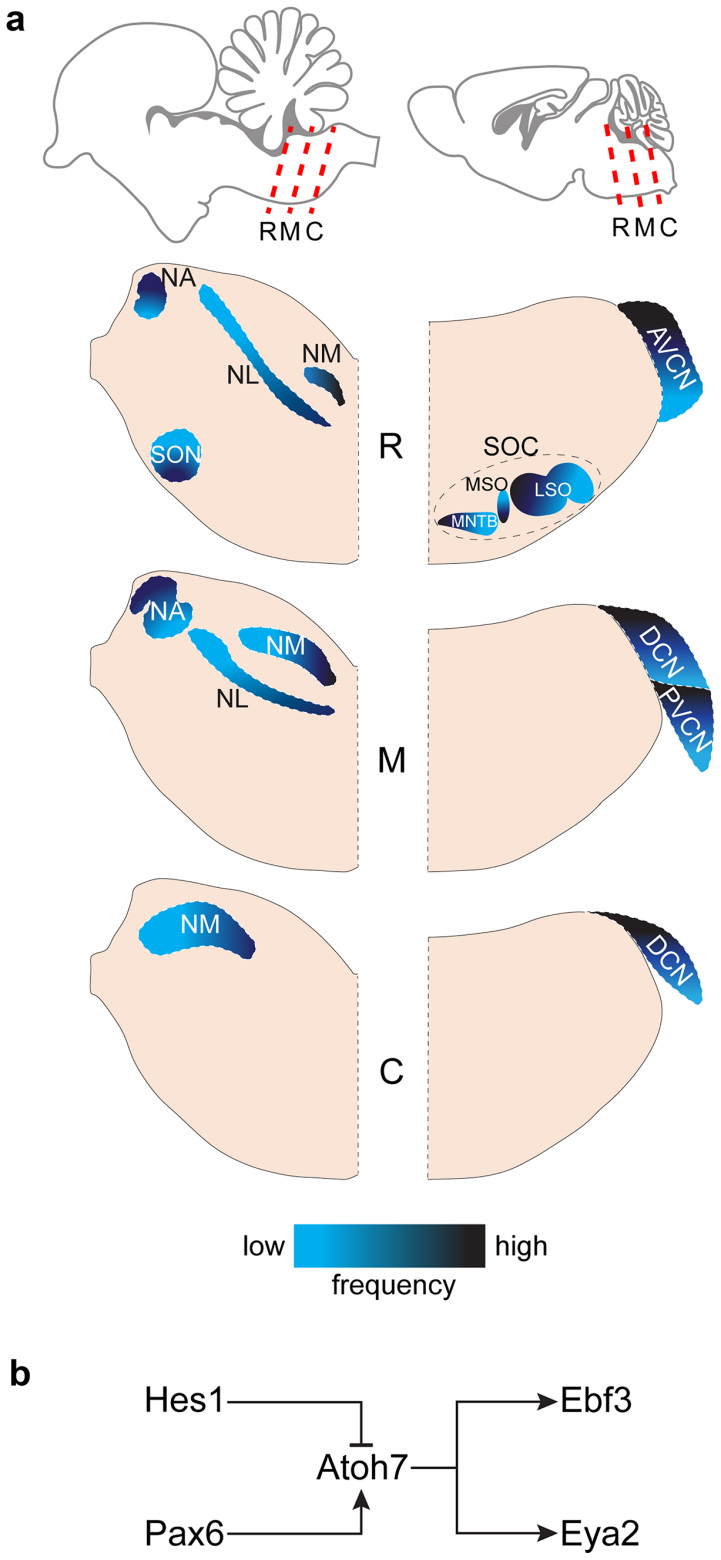
Auditory nuclei in the chicken and mouse hindbrain. (**a**) Top: Schematic views of sagittal section of chicken (left) and mouse (right) brain with correspondent locations (red dashed lines) of representative rostral (R), middle (M), and caudal (C) coronal sections. Below: A simplified depiction of the auditory hindbrain nuclei in coronal sections studied here in chicken (left) and mice (right). The chicken auditory nuclei include the nucleus angularis, nucleus magnocellularis, nucleus laminaris, and the superior olivary nucleus. The mouse auditory nuclei include the dorsal cochlear nucleus, anterior ventral cochlear nucleus, the posterior ventral cochlear nucleus, and the superior olivary complex (SOC). The SOC is composed of several subnuclei, where we depict here the major three: the medial nucleus of the trapezoid body, the medial superior olive, and the lateral superior olive. Darker and lighter blue represent higher and lower frequency regions, respectively. The tonotopic gradients are simplified according to how they can be viewed on a coronal slice. AVCN, anteroventral cochlear nucleus; C, caudal; D, dorsal; DCN, dorsal cochlear nucleus; LSO, lateral superior olive; M, middle; MNTB, medial nucleus of the trapezoid body; MSO, medial superior olive; NA, nucleus angularis; NM, nucleus magnocellularis; NL, nucleus laminaris; PVCN, posteroventral cochlear nucleus; SOC, superior olivary complex; SON, superior olivary complex; V, ventral. (**b**) Diagram depicting the simplified *Atoh7* gene regulatory pathway as suggested by (Gao et al. 2014) operating in retinal progenitor cells. *Hes1* and *Pax6* are regulators of *Atoh7* expression as an inhibitor and activator, respectively. *Ebf3* and *Eya2* are regulated (activated) by *Atoh7*. All abbreviations also apply to remaining figures

To compare the gene expression pattern of the *Atoh7* centered GRN module in the developing amniote auditory brainstem, we performed in situ hybridization of *Atoh7* and its upstream regulator genes *Hes1* and *Pax2* as well as *Ebf3* and *Eya2* as two downstream located genes in mice and chicken (Fig. 1). In the mouse, we focused on the cochlear nucleus complex with its three subdivisions dorsal cochlear nucleus (DCN), and posterior and anterior ventral cochlear nucleus (PVCN, AVCN) as the sole structure of *Atoh7* expression in the auditory hindbrain (Fig. 1a) (Saul et al. 2008). Note that the other four genes are expressed in the superior olivary complex (suppl. Fig. 1). This, however, was not followed up, as their expression there is not part of an *Atoh7* centered GRN, as this auditory structure does not express *Atoh7* at any time during development (Saul et al. 2008). As the *Atoh7* centered GRN has not been studied in any of the chicken auditory nuclei, the entire avian auditory hindbrain was scrutinized, i.e. the second order nucleus angularis (NA) and nucleus magnocellularis (NM) as well as the third order nucleus laminaris (NL) and the superior olivary nucleus (SON) (Fig. 1a). Expression patterns were determined at two time points, an immature, prehearing stage (postnatal day (P) 4 in mice and embryonic day (E) 13 in chicken) and the mature stage after hearing onset (P30 in mice and P14 in chicken).

### Expression pattern in the mouse cochlear nucleus complex at P4

**Fig. 2:**
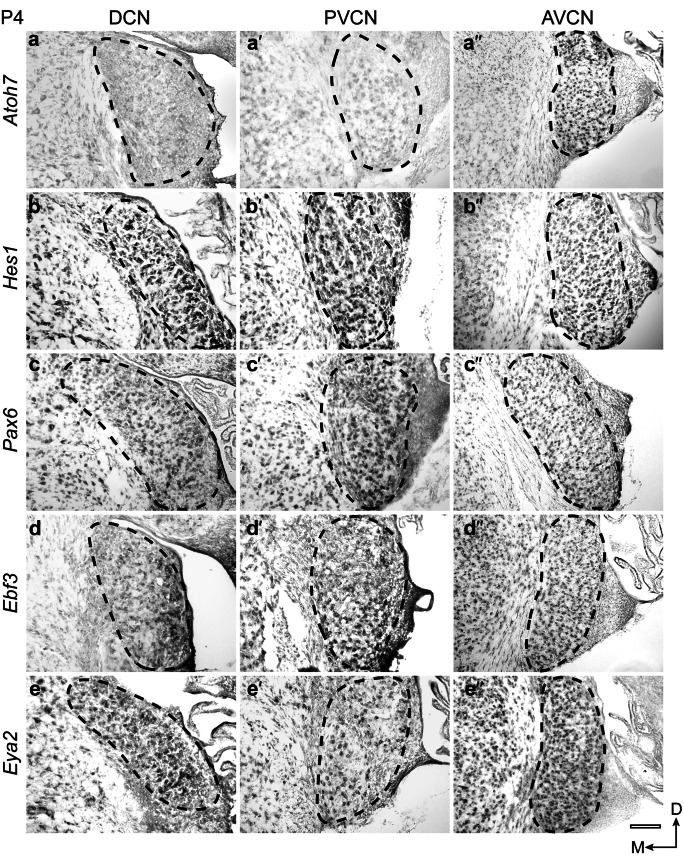
Spatiotemporal expression of *Atoh7*, *Hes1*, *Pax6*, *Ebf3*, and *Eya2* in the mouse cochlear nuclei at P4. On-slide RNA in situ hybridization on coronal brain sections. *Atoh7* (**a**–**a´´**) expression is restricted to the AVCN. *Hes1* (**b**–**b´´**), *Pax6* (**c**–**c´´**), *Ebf3* (**d1**–**d´´**), and *Eya2* (**e**–**e´´**) showed expression in all three cochlear nuclei. All transcription factors, except *Atoh7*, were expressed broadly in the DCN, PVCN, and AVCN, with minor differences in the intensity and breadth of expression. Scale bar: 100 μm

Analysis of *Atoh7* expression at P4 confirmed that its expression is confined to the AVCN within the auditory brainstem (Fig. 2a´´) (Saul et al. 2008). In contrast, the other four genes of this GRN module, *Hes1* (Fig. 2b-b´´), *Pax6* (Fig. 2c-c´´), *Ebf3* (Fig. 2d-d´´) and *Eya2* (Fig. 2e-e´´) were expressed in all three cochlear nuclei, i.e. the DCN, PVCN, and AVCN, with minor differences in the intensity and breadth of expression. For example, *Hes1* showed a slightly stronger expression in the DCN and PVCN compared to the AVCN (Fig. 2b-b´´), and *Ebf3* was expressed in the DCN mainly in the fusiform layer (Fig. 2d).

### Expression pattern in the mouse cochlear nucleus complex at P30

**Fig. 3:**
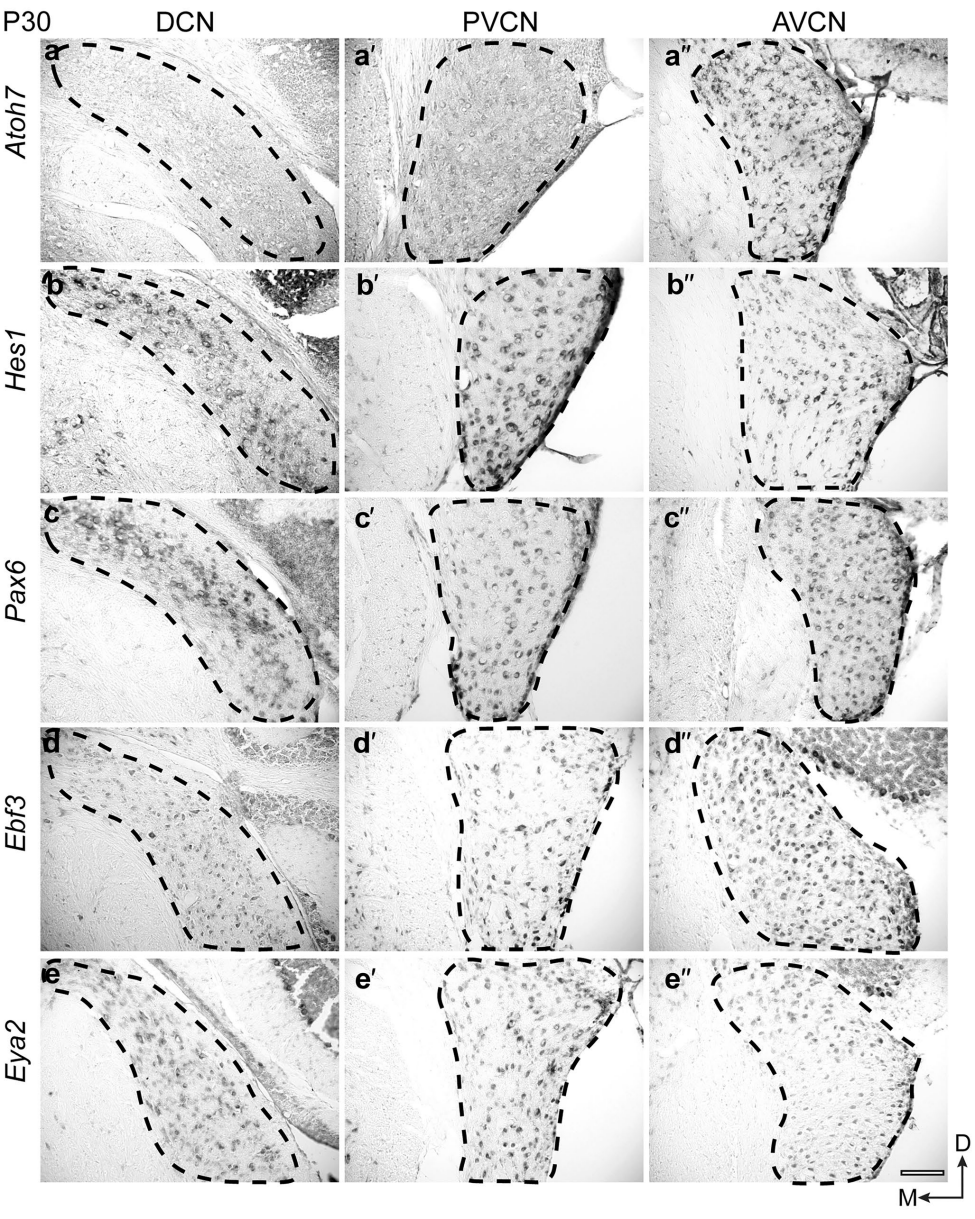
Spatiotemporal expression of *Atoh7*, *Hes1*, *Pax6*, *Ebf3*, and *Eya2* in the mouse cochlear nuclei at P30. On-slide RNA in situ hybridization on coronal brain sections. *Atoh7* (**a**–**a´´**) expression is restricted to the AVCN. *Hes1* (**b**–**b´´**), *Pax6* (**c**–**c´´**), *Ebf3* (**d**–**d´´**), and *Eya2* (**e**–**e´´**) showed expression in all three cochlear nuclei. All transcription factors, except *Atoh7*, were expressed broadly in the DCN, PVCN, and AVCN, with minor differences in the intensity and breadth of expression. Scale bar: 100 μm

In the adult mouse, *Atoh7* expression was again confined to the AVCN with no expression in the DCN or PVCN (Fig. 3a-a´´). *Hes1* (Fig. 3b-b´´), *Pax6* (Fig. 3c-c´´), *Ebf3* (Fig. 3d-d´´), and *Eya2* (Fig. 3e-e´´) showed again broad expression across all three cochlear subdivisions with some minor differences. *Ebf3* expression was weaker in the DCN than in the VCN and did not mark the fusiform layer as in P4 (Figs. 2d, 3d, d´), whereas *Eya2* expression was weak in the AVCN compared to the other two cochlear subdivisions (Fig. 3e-e´´). Furthermore, both *Hes1* and *Pax6* expression in the DCN was most prominent in the fusiform layer (Figs. 3b, c).

### Expression pattern in chicken auditory hindbrain nuclei at E13

**Fig. 4:**
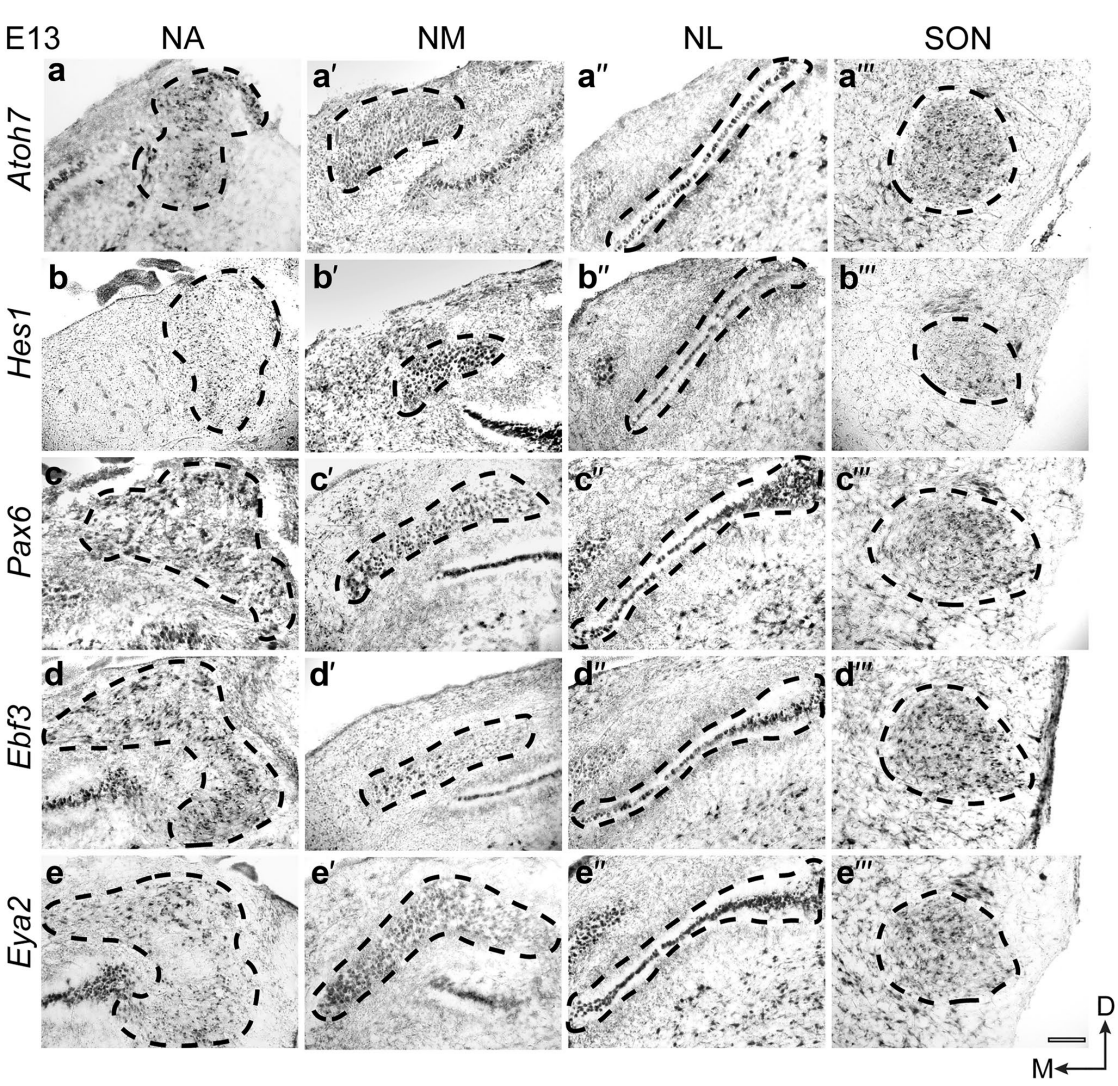
Spatiotemporal expression of *Atoh7*, *Hes1*, *Pax6*, *Ebf3*, and *Eya2* in the chicken auditory hindbrain at E13. On-slide RNA in situ hybridization on coronal brain sections having rostral, middle, and caudal regions of the NM. *Atoh7* (**a**–**a´´´**), *Hes1* (**b**–**b´´´**), *Pax6* (**c**–**c´´´**), *Ebf3* (**d**–**d´´´**), and *Eya2* (**e**–**e´´´**) showed expression in all four auditory nuclei. All transcription factors were expressed broadly in the NA, NM, NL, and SON with minor differences in the intensity and breadth of expression; except for *Atoh7* in the NM and *Hes1* in the SON, which had minimal and selective expression. Scale bar: 100 μm

In the embryonic chicken (E13) hindbrain, *Atoh7* was broadly expressed in the NA, NL, and SON, but rather absent in the rostral NM regions (Fig. 4a-a´´´). *Hes1* showed a lower expression in the NA compared to the other four genes(4b). In the NM and NL, it was strongly expressed, whereas in the SON it showed a selective expression in the ventral part (prospective high frequency processing neurons) (Fig. 4b´-b´´´). *Pax6* was strongly expressed in NA, NL, and SON, and showed a graded expression in the NM, with higher expression in the medial region compared to the lateral region (prospective low frequency processing neurons) (Fig. 4c-c´´´). *Ebf3* and *Eya2* were broadly expressed in the NA, NL, and SON, with slight differences in the intensity and breadth of expression. In the NM, *Ebf3* showed a selective expression restricted to the medial region of the nuclei, and *Eya2* showed a gradient expression with high expression in the medial area (Figs. 4d´, e´).

### Expression pattern in chicken auditory hindbrain nuclei at P14

**Fig. 5:**
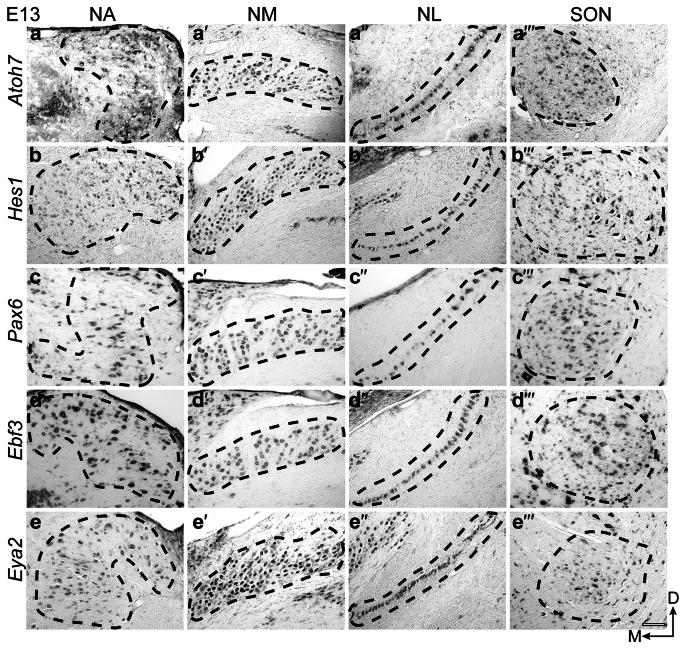
Spatiotemporal expression of *Atoh7*, *Hes1*, *Pax6*, *Ebf3*, and *Eya2* in the chicken auditory hindbrain at P14. On-slide RNA in situ hybridization on coronal brain sections. *Atoh7* (**a**–**a´´´**), *Hes1* (**b**–**b´´´**), *Pax6* (**c**–**c´´´**), *Ebf3* (**d**–**d´´´**), and *Eya2* (**e**–**e´´´**) showed expression in all four auditory nuclei. All transcription factors were expressed broadly in the NA, NM, NL, and SON with minor differences in the intensity and breadth of expression; except for evident weak expression of Hes1 and Eya2 in NA and selective expression of Eya2 in SON. Scale bar: 100 μm

In the adult chicken (P14), *Atoh7* was broadly expressed in all auditory hindbrain nuclei, i.e. NA, NM, NL, and SON. *Hes1* and *Pax6* also showed a broad expression in all chicken auditory nuclei, with slightly lower intensity and breadth of expression of *Hes1* in the NA (Figs. 5b, c). *Ebf3* showed a higher expression in NA compared to the other auditory nuclei (5d-d´´´), whereas *Eya2* showed strong expression in the NM and NL and weak expression in the NA and SON (Figs. 5e-e´´´).

### Differential spatial gene expression in the chicken NM at E13

**Fig. 6:**
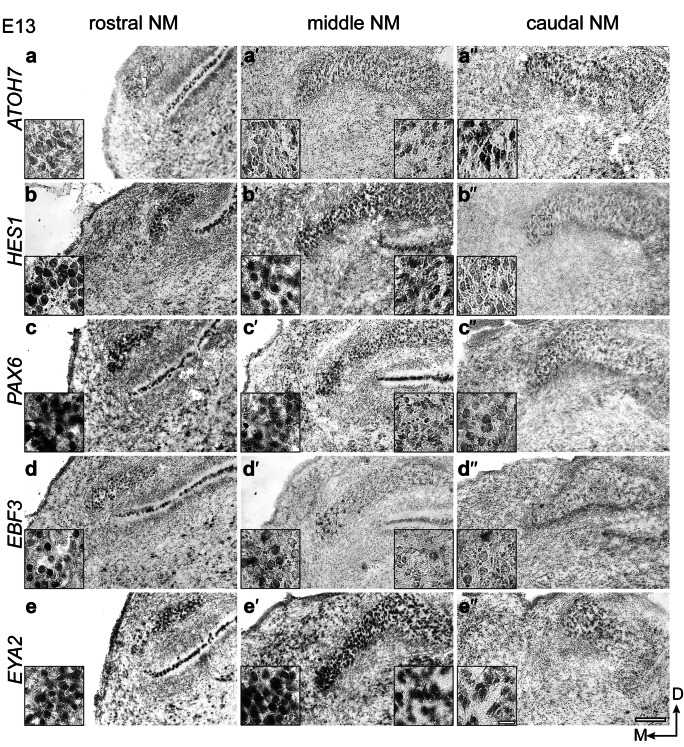
Spatiotemporal expression of *Atoh7*, *Hes1*, *Pax6*, *Ebf3*, and *Eya2* in different rostro-caudal regions of the chicken NM at E13. On-slide RNA in situ hybridization on coronal brain sections. *Atoh7* (**a**–**a´´**) expression was nearly absent in rostral region, with moderate expression in the middle region, and a stronger expression in the caudal region of NM. *Hes1* (**b**–**b´´**) showed a stronger expression in the rostral region, a milder expression in the middle region, and very low and localized expression in the medial side of the caudal region of NM. *Pax6* (**c**–**c´´**) showed a high expression in the rostral region, a milder expression in the middle region forming a gradient with higher expression in the medial side than the lateral side, and a similar gradient in the caudal region of NM with lower intensity. *Ebf3* (**d**–**d´´**) showed a higher expression in the rostral region with absence in few cells and a selective expression in middle region with few positive cells in the medial side, but expression was nearly absent in the caudal region of NM. *Eya2* (**e**–**e´´**) showed a strong expression in the rostral region, a gradient expression in the middle region with higher expression in the medial side compared to the lateral side, and a similar but milder gradient in the caudal region of the NM. Scale bar: 100 μm in 20 × images; 20 µm in 60 × inset images

Our in situ analysis indicated differential spatial expression of all five genes in the NM at E13. To analyze their expression in more detail, we chose slices representing rostral, middle, and caudal regions of the NM to probe the observed differential expression. *Atoh7* was almost absent in the rostral region, moderately expressed in the middle region, and strongly expressed in the caudal NM (Fig. 6a-a´´). *Hes1* showed an opposite pattern as seen with *Atoh7*. Expression was broad and high in the rostral region, moderate in the middle region and low in the caudal region (Fig. 6b-b´´). *Pax6* showed a clear graded expression across the rostro-caudal axis. In the rostral region, its expression was highest and broadest, and in the middle and caudal regions a gradient expression was observed with higher expression towards the medial side (Fig. 6c-c´´). *Ebf3* displayed a very selective expression. In the rostral region, most cells expressed *Ebf3*, whereas in the middle and caudal regions, very few medially located cells expressed *Ebf3* (Fig. 6d-d´´). Finally, *Eya2* showed a gradient expression pattern similar to *Pax6* with high expression in the future high frequency area (Fig. 6e-e´´). The difference in expression intensities between NM regions is also visualized in higher magnification inset images (Fig. 6).

**Fig. 7:**
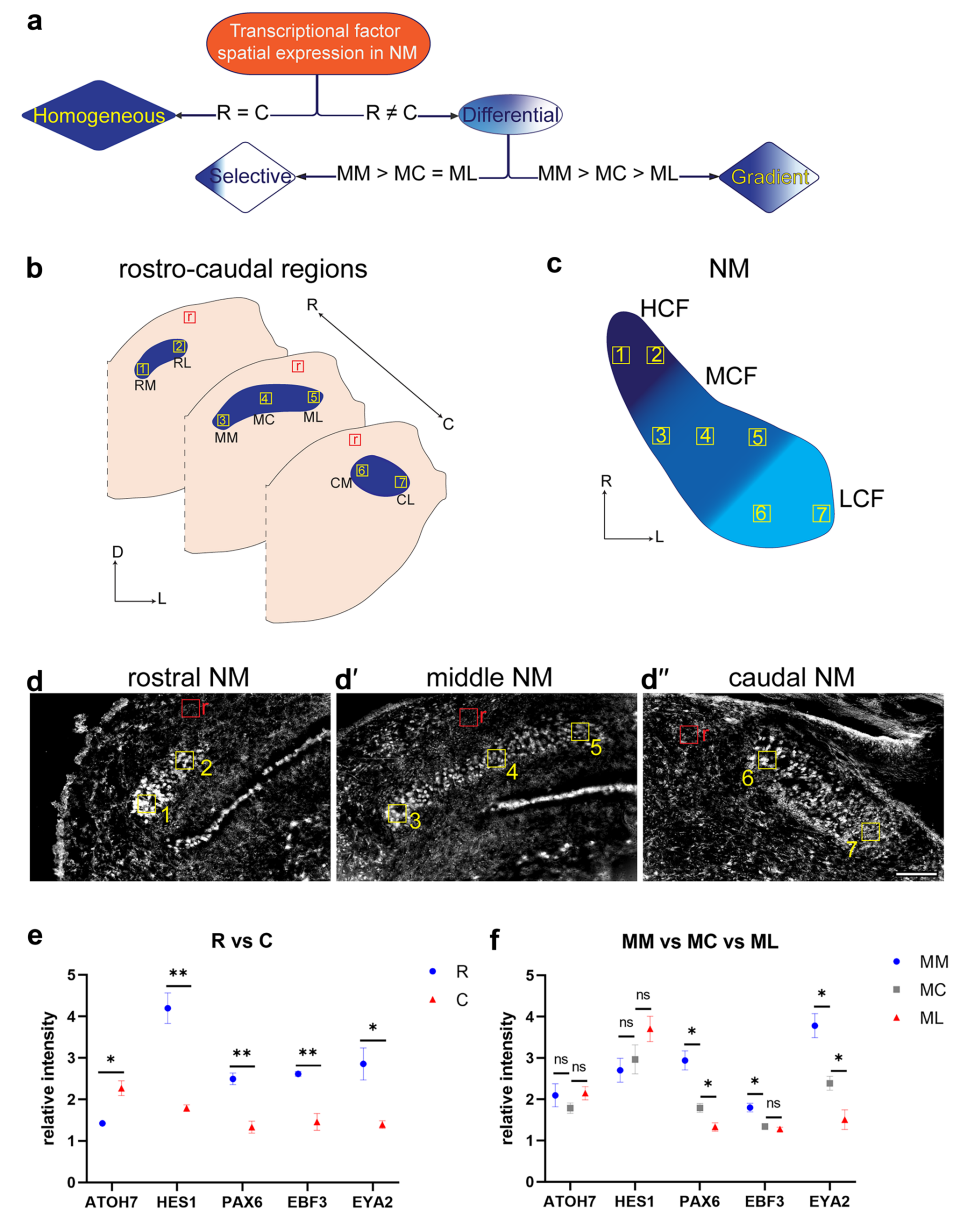
Quantitative spatial expression intensity analysis of transcription factors in different rostro-caudal regions of the NM at E13. (**a**) Decision tree used for categorizing different expression patterns. (**b**) Scheme of different coronal slice at rostral, middle, and caudal planes of the NM, with seven different selected regions of interest (ROIs) for intensity measurements. Each ROI presented here was analyzed in at least three different slices pertaining to three different embryonic chicks (**c**) Superimposition of the seven selected regions of interest on a schematic dorsal view of NM illustrating the tonotopic organization of high, middle, and low CF neurons along the rostro-medial to caudolateral axis. (**d**-**d´´**)Representative inverted grayscale images used for intensity images, showing numbered ROIs and corresponding reference regions (r) in rostral, middle, and caudal NM slices. The mean gray value of each ROI was measured and normalized to the reference region, and two to four replicates were measured for each ROI. (**e**) Comparison between the relative intensities of the transcription factors expression in rostral regions (average of RM and RL) with caudal regions (average of CM and CL) of the NM. All transcription factors expression displayed a significant difference between rostral and caudal NM regions, designating their expression pattern as “differential”. Only *Atoh7* was expressed higher at the caudal regions of the NM compared to rostral regions, whereas the other four transcription factors were expressed higher in the rostral regions when compared to the caudal regions of the NM. (**f**) Comparison between the relative intensities in expression in the MM with MC, and in the MC with ML regions of the middle NM. *Pax6* and *Eya2* fit the pattern designated as “gradient” with MM > MC > ML, and *Ebf3* fits the pattern designated as “selective” with MM > MC = ML. Abbreviations: CL, caudo-lateral; CM, caudo-medial; MC, middle-central; ML, middle-lateral; MM, middle-medial; RL, rostro-lateral; RM, rostro-medial. Statistics: Error bars represent SEM. ns, not significant; *, p value < 0.05; **, p value < 0.01; ***, p value < 0.001

To quantify this visual impression, we performed a quantitative expression analysis to categorize the different spatial patterns in the embryonic NM (Fig. 7). We defined different spatial expression patterns according to the decision tree presented in Fig. 7a. This analysis demonstrated the differential expression of all five transcription factors between rostral and caudal regions of the E13 NM. *Atoh7* is significantly higher expressed in the caudal NM region than in the rostral NM region (Fig. 7e). On the contrary, *Hes1*, *Pax6*, *Ebf3*, and *Eya2* are significantly higher expressed in the rostral NM region than in the caudal region (Fig. 7e). Further analysis revealed different expression patterns in the middle part of the NM, when this region was subdivided into a medial (MM), central (MC), and lateral (ML) part. Both *Atoh7* and *Hes1* showed no significant difference in expression in this region of the NM (Fig. 7f). In contrast, *Pax6* and *Eya2* showed a gradient pattern, with a significantly higher expression in the MM region compared to the MC region and a significantly higher expression in the MC region compared to the ML region (Fig. 7f). *Ebf3* had a selective expression pattern with a significantly higher expression in the MM compared to the MC and no significant difference between MC and ML (Fig. 7f).

## Discussion

The results of our cross-species comparative expression study of transcription factors of an *Atoh7* centered GRN can be summarized in three major points: 1) Identification of the first transcription factor with strikingly different expression between the mouse and chicken auditory hindbrain. 2) Broad expression of retinal gene regulatory network components in the tetrapod auditory hindbrain. 3) Differential spatial expression of all five analyzed genes in the embryonic NM.

### Identification of the first transcription factor with strikingly different expression patterns between the mouse and chicken auditory hindbrain

The restricted expression of the murine *Atoh7* gene and the broad expression of its chicken orthologue identifies for the first time a GRN component with strikingly different expression pattern in the auditory hindbrain of the two species. One explanation for this difference might be that our chosen postnatal time points in mice (P4, P30) missed earlier *Atoh7* expression in the mouse hindbrain. However, a previous analysis which sampled across the entire development by analyzing adult *Atoh7-lacZ* reporter mice did also not detect *Atoh7* expression outside bushy cells of the AVCN (Saul et al. 2008). Thus, *Atoh7* expression in the mammalian auditory hindbrain is highly restricted. The shared expression of this gene in second order glutamatergic neurons of the VCN and NM therefore point to a close genetic relationship of these two cell types which is supported by their shared origin from an Atoh1 transcription factor lineage (Fujiyama et al. 2009; Maricich et al. 2009; Lipovsek and Wingate 2018).

We note that the expression of the upstream transcriptional activator *Pax6* and the downstream transcription factors *Efb3*, and *Eya2* in the PVCN and DCN in the absence of Atoh7 point to a different GRN logic in the mammalian hindbrain compared to the retinal *Atoh7* GRN module. In the chicken, the situation is more complex. All five genes were expressed in all nuclei, which is compatible with their genetic linkage within a common GRN. Further studies, however, are needed to corroborate their co-expression in the same cells. The inverse expression patterns of *Atoh7* and its transcriptional repressor *Hes1* in the embryonic NM fit the genetic linkages observed in the retina, where deletion of *Hes1* increases *Atoh7* expression (Lee et al. 2005). In contrast, the inverse expression gradients of *Atoh7* and its transcriptional activator *Pax6* or the *Atoh7* downstream targets *Efb3* and *Eya2* in the embryonic NM disagree with the results obtained in the mammalian retina.

The differences observed between mouse and chicken are in agreement with reported species-specific differences in the upstream regulation of *Atoh7* and downstream targets in the mouse and chicken retina (Skowronska-Krawczyk et al. 2009). The vertebrate *Atoh7* gene contains two conserved upstream regulatory domains which show different occupancy patterns by the transcription factor neurogenin 2. In the chicken, neurogenin 2 strongly binds to the proximal domain which is not the case in the mouse retina, despite conservation of the regulatory *Atoh7* DNA sequence (Skowronska-Krawczyk et al. 2009). This, together with a species-specific positive feedback of Atoh7 on its own expression results in a tenfold higher *Atoh7* expression during early development of the chicken retina compared to the mouse. As a consequence, several targets of *Atoh7* showed higher expression levels in the chicken retina compared to the mouse, including the neuronal growth cone associated genes stathmin-2 and Robo2.

Overall, the observed differences in expression of the *Atoh7* GRN module between the mammalian and avian auditory system provide molecular support for an independent evolution of second-order nuclei. This also applies to the mammalian medial superior olive and the avian NL, two functionally and anatomically similar third order nuclei. Both structures act as coincidence detectors and process interaural time differences with inhibitory neurotransmission serving an important role therein. Consequently, they were considered to be homologous for many years. Yet, the different presynaptic inhibitory neurotransmitters (MSO: glycine, NL: GABA), their different action (MSO: hyperpolarization, NL: depolarization), time scale: (MSO: precise temporal integration; NL: decoupled from phase-locked inhibition) and coding strategy (MSO: population code, NL: place code) argue for a convergent evolution of the two nuclei (Grothe and Pecka 2014). The absence of *Atoh7* in the medial superior olive (Saul et al. 2008) and its presence in the NL (Figs. 4, 5) add genetic evidence to this view of convergent evolution. The shared expression of the other GRN components, i.e. *Hes1*, *Pax6*, *Ebf3*, and *Eya2* (this study), *Hoxd1*, *Mab21l2*, *Meis2* and Mitf (Pawlik et al. 2016), as well as eleven microRNAs (Pawlik et al. 2016; Saleh and Nothwang 2021) in the amniote auditory hindbrain point also to mechanisms of parallel evolution which might be facilitated by the highly conserved genetic architecture of rhombomeres across vertebrates (Tümpel et al. 2009; Parker et al. 2016; Pascual-Anaya et al. 2018). This likely results in the use of similar GRN components to create interspecies neuronal diversity across different vertebrates. This evolutionary conserved neural genoarchitecture of the developing hindbrain facilitates development of homoplasious structures by parallel evolution using homologous genes, reflecting the deep homology of these amniote structures (Gray 2008; Nothwang 2016). Summarizing all available data, it is thus likely that the vertebrate auditory hindbrain represents a mosaic of parallel and convergent evolution. The precise breakdown of the individual contributions of both mechanisms requires detailed functional analyses of the exact role of gene regulatory network components for a given feature.

The rich repertoire of genetically modified mice and the availability of CRISPR/Cas9 and shRNA techniques to manipulate gene expression in both mice and chicken pave the way for this kind of comparative analysis. An instructive example is provided by recent studies on the function of the fragile X mental retardation protein FMRP in both the mouse and chicken auditory brainstem. In mice lacking FMRP, expression of the presynaptic proteins VGLUT1 and Syt1 was altered in the large calyx of Held synapse between bushy cells in the cochlear nucleus and neurons of the medial nucleus of the trapezoid body during a period of convergence of multiple pre-calyxes into single calyx terminals (Yu and Wang 2022). In the chicken, downregulation of the protein resulted in smaller size and abnormal morphology of individual presynaptic endbulbs in the NM, as well as perturbed connectivity between NM and NL neurons such as perturbed axonal pathfinding, delayed midline crossing, excess branching of neurites, and axonal targeting errors during the period of circuit development (Wang et al. 2018, 2020). These data suggest an important function of FMRP in auditory circuit formation in both vertebrate groups. Conclusion on an identical function, however, has to awaits detailed analysis of corresponding circuits.

### Shared expression of retinal GRN components in the tetrapod auditory brainstem: similar but different

All five genes analyzed add to the list of GRN components with expression in both the visual and auditory system such as the miRNA-183 family (Banks et al. 2020; Dambal et al. 2015) or *MafB* (Hamada et al. 2003; Marrs et al. 2013). On first glance, this lends support on the molecular level to the recently proposed notion of unifying principles in neural circuit formation (Sitko and Goodrich 2021). However, detailed functional analyses are required to draw any conclusion on similar genetic mechanisms operating across modalities, as conflicting data exist.

The co-option of genes to multiple circuits might reflect similar demands. Notably, retinal ganglion cells, VCN bushy cells and the NM neurons share various traits. All three are second-order glutamatergic excitatory neurons, receive signals from bipolar neurons, i.e. retinal bipolar and auditory nerve cells, respectively, and display a role in the contralateral representation of visual and auditory space in the higher-order cortex regions, via decussating fibers in the optic chiasm and the brainstem, respectively (Saul et al. 2008).

The shared genetic factors might contribute to these similarities. Indeed, analyses in transgenic mice indicate both common and distinct functions of genes expressed across different sensory systems. In retinal ganglion cells, *Atoh7* is required for survival and correct axonal guidance (Brodie-Kommit et al. 2021), whereas in the auditory brainstem, *Atoh7* deficient bushy cells are still present but reduced in size (Saul et al. 2008). Despite this mild morphological phenotype, alterations in auditory brainstem responses were observed that suggest disturbed connection with the target neurons (Saul et al. 2008). This indicates a projection deficit as seen in retinal ganglion cells. Nevertheless, detailed analysis in the auditory brainstem is required for firm conclusion. Overlapping and distinct functions are also observed for the miR-183 family, consisting of miR-183, miR-96, and miR-182. miR-183/96 double knockout mice demonstrate impaired development both in hair cells and photoreceptors. In the auditory system, abnormal hair cell stereocilia bundles and reduced numbers of inner hair cell synapses were observed (Lewis et al. 2020) and in the visual system, defects in photoreceptor maturation, maintenance, and function such as polarization defect of the cones and photoreceptor degeneration (Xiang et al. 2017). Furthermore, ablation of the third family member, miR-182, has not been associated with a developmental defect in the cochlea (Lewis et al. 2020) nor the retina (Jin et al. 2009). Contrary to these similarities, point mutations in miR-96 result in hearing loss (Lewis et al. 2009; Mencia et al. 2009), whereas vision is unaffected (Mencia et al. 2009; Xu et al. 2020). These examples suggest that the same gene can execute similar but also sensory system-specific functions, likely reflecting the embedding into different genetic networks. A major task will therefore be to decipher in detail the function of shared genes in sensory systems differing either in modality or species, and to define their precise role and thus the extent of unifying genetic mechanisms in neuronal circuit development, maintenance, and function. Our identification of *Atoh7*, *Hes1*, *Pax6*, *Ebf3*, and *Eya2* add attractive candidates to these studies across sensory modalities and organisms.

### Differential spatial expression in the embryonic NM

We observed a differential spatial expression pattern for all five genes in the NM at E13 along the prospective tonotopic axis. *Atoh7* was stronger expressed in the caudal region of NM harboring the prospective low characteristic frequency neurons, and nearly absent in the rostro-medial pole of the NM containing the prospective high characteristic frequency neurons. The other four genes, *Hes1*, *Pax6*, *Ebf3*, and *Eya2*, showed a higher expression in the rostral region, with *Hes1* and *Ebf3* nearly absent in the caudolateral pole. This differential expression patterns add to the previously reported graded expression of nine miRNAs in the E13 NM with high expression in the rostro-medial area (Saleh and Nothwang 2021). One explanation of the differential expression of the five transcription factors in the NM is their regulation by those miRNAs. However, none of the studied miRNAs shows an opposite expression gradient compared to these transcription factors, except for *Atoh7*. In agreement, none of the transcription factors were identified as targets of these miRNAs (Saleh and Nothwang 2021). This clearly refutes this hypothesis. Another explanation is that the differential expression of these transcription factors is driving the graded expression of those miRNAs. Finally, the graded expression of the miRNAs and the transcription factors might occur independently from each other. In any case, similar to the miRNAs, the graded expression of these five transcription factors in the multisegmental NM might reflect rhombomere-specific action of GRNs (Saleh and Nothwang 2021).

The mid-embryonic stage around E13 is characterized in the NM by many developmental graded processes, including cell growth and death, retraction of transient dendrites, formation of endbulbs of held, and the onset of postsynaptic responses (Rubel and Parks 1988). During development, these gradients proceed from the rostro-medial pole (the prospective high-frequency region) towards the caudolateral pole (the prospective low-frequency region) (Rubel and Parks 1988). It is tempting to link the graded expression of the five transcription factors to these developmental processes. One prominent progression results in a steep spatial gradient in NM dendritic numbers, with neurons at rostro-medial region having about two dendrites, and cells in caudolateral region having an average of nine dendrites. Notably, *Stmn2*, *Snap25*, and *Robo2*, which are involved in the development of dendritic arbors and axons were shown to be positively regulated by *Atoh7* in a dose-dependent manner (Skowronska-Krawczyk et al. 2009). This matches our observation of *Atoh7* being concentrated in the caudolateral region of NM, whose neurons (low-frequency) are known to have longer dendrites with intricate branching and longer axonal stretches (Akter et al. 2020; Kuba and Ohmori 2009; Wang et al. 2017). Future studies should therefore analyze the expression and function of *Stmn2*, *Snap25*, and *Robo2* in the developing NM.

In conclusion, our data add genetic evidence for independent evolution of the amniote auditory hindbrain. First, we identified a marked difference in the expression pattern of a transcription factor between the mammalian and avian auditory hindbrain. Second, the striking presence of graded expression of the large majority of GRN components analyzed so far in the avian auditory hindbrain and its absence in the mammalian auditory system indicate different developmental principles for organizing tonotopic features between the mammalian and avian auditory system. Yet, the development of homoplasious traits in the amniote auditory hindbrain is likely based on co-option of many genetic factors, due the evolutionary highly conserved genoarchitecture present in rhombomeres. This close genetic relationship might especially apply to bushy cells and the neurons of the nucleus magnocellularis as the only amniote hindbrain neurons sharing *Atoh7* expression. These two cell types might therefore represent sister cell types (Arendt et al. 2016). Our results add further evidence to the notion that the NM acts as a central organizer of tonotopic features in the avian auditory hindbrain. Finally, the expression of all five transcription factors in the auditory system validates our approach to exploit information from other sensory modalities to generate entry points to decipher genetic networks operating in the auditory system and support the view of shared genetic mechanisms in neuronal circuit formation across modalities and species.

## Supplementary Information

Below is the link to the electronic supplementary material.

Supplementary file1 (TIF 10555 KB)
