# Supplementary figures and images for: Comparative expression analysis of the Atoh7 gene regulatory network in the mouse and chicken auditory hindbrain
Source: Cell Tissue Res. 2023 Mar 24;392(3):643–58. doi: 10.1007/s00441-023-03763-9 (PMC10235153; doi:10.1007/s00441-023-03763-9)

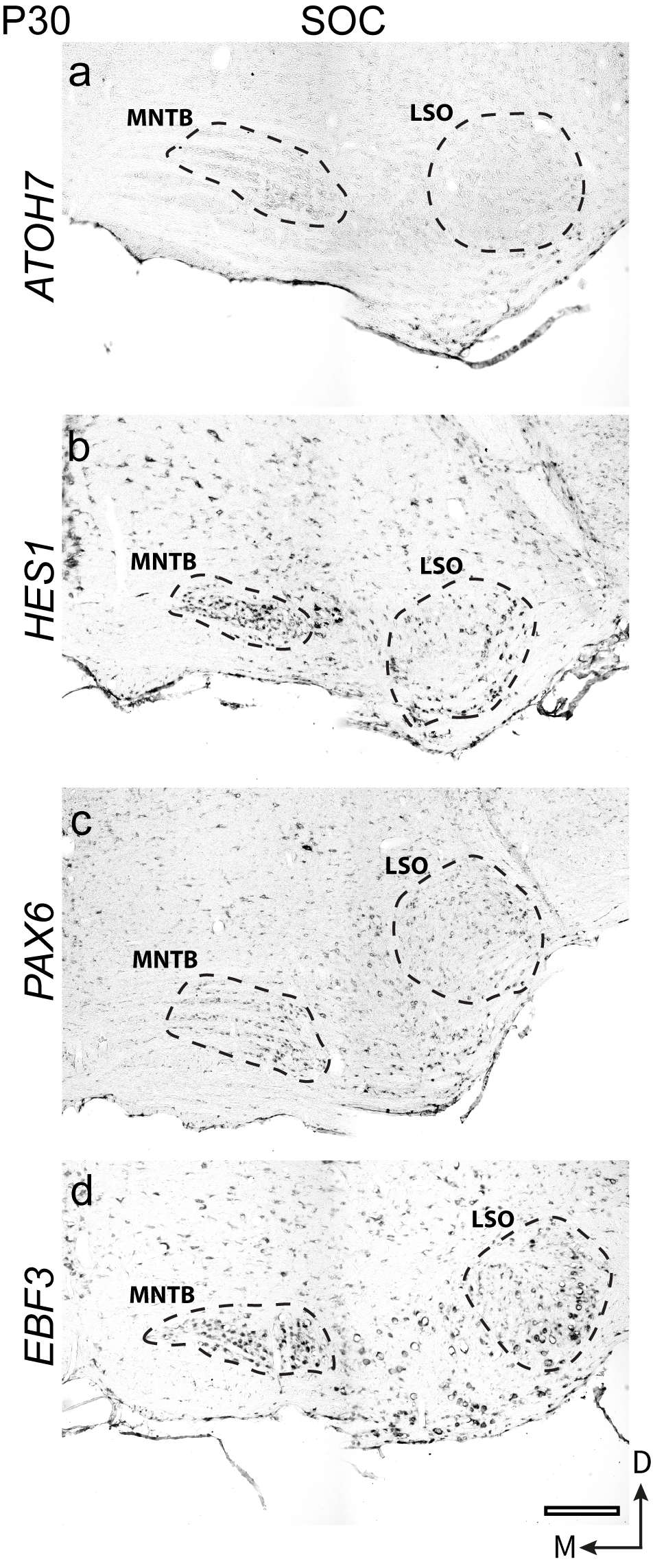

Supplement: Supplementary file 1 — Supplementary file1 (TIF 10555 KB) [file 441_2023_3763_MOESM1_ESM.tif]
